# The discovery of cholera - like enterotoxins produced by *Escherichia coli* causing secretory diarrhoea in humans

**Published:** 2011-02

**Authors:** R. Bradley Sack

**Affiliations:** *Johns Hopkins University, Bloomberg School of Public Health, Baltimore, Maryland, USA*

**Keywords:** Cholera, diarrhoea, enterotoxin, non-cholera diarrhoea

## Abstract

Non-vibrio cholera has been recognized as a clinical entity for as long as cholera was known to be caused by *Vibrio cholerae*. Until 1968, the aetiologic agent of this syndrome was not known. Following a series of studies in patients with non-vibrio cholera it was found that these patients had large concentrations of *Escherichia coli* in the small bowel and stools which produced cholera toxin-like enterotoxins, and had fluid and electrolyte transport abnormalities in the small bowel similar to patients with documented cholera. Furthermore, these patients developed antibodies to the cholera-like enterotoxin. Later studies showed that these strains, when fed to volunteers produced a cholera-like disease and that two enterotoxins were found to be produced by these organisms: a heat-labile enterotoxin (LT) which is nearly identical to cholera toxin, and a heat-stable enterotoxin (ST), a small molecular weight polypeptide. *E. coli* that produced one or both of these enterotoxins were designated enterotoxigenic *E. coli* (ETEC). ETEC are now known not only to cause a severe cholera-like illness, but to be the most common bacterial cause of acute diarrhoea in children in the developing world, and to be the most common cause of travellers’ diarrhoea in persons who visit the developing world.

## Scope of this review

The review covers the following:

1. The development of the rabbit ileal loop (RIL) method by Dr S.N. De in 1953.
2. The use of the RIL to study live cultures of *Escherichia coli* isolated from stools of patients with cholera- like disease.
3. The clinical characterization of non-vibrio cholera during an unusual epidemic in 1963.
4. The demonstration that *E. coli* produced a cholera toxin-like enterotoxin in patients with cholera-like disease in 1968.
5. The rapid development of studies in the first 5 years after the findings of enterotoxigenic *E. coli* (ETEC) was published. These include: (*i*) the recognition that both heat-labile and heat-stable enterotoxins (LT and ST) were separate enterotoxin, (*ii*) the recognition of ETEC as an important pathogen in parts of the U.S., (*iii*) the discovery of pili (fimbria) as a critically important virulence factor for ETEC, and (*iv*) the important role of ETEC in the diarrhoea of travellers.

## Work of Dr S.N. De

De developed the RIL in 1953^1^, and used it to study *Vibrio cholerae* infections and to discover the cholera toxin in 1959^2^. In 1956, he also used it to study *E. coli* isolated from the stools of adult patients with cholera-like symptoms but from whom *V. cholerae* could not be found^3^. He used the RIL to test a live single colony culture of *E. coli* from each of 20 patients with non-vibrio cholera and 20 controls. About 65 per cent of the patients and 15 per cent of the controls gave a positive RIL. These data suggested that these *E. coli* may be the cause of the diarrhoea. Unfortunately, he did not test the cell-free filtrates of these *E. coli*.

**Fig. 1:**
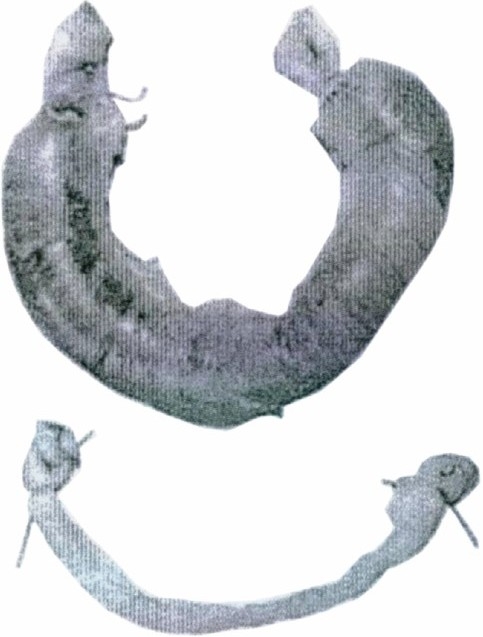
Gross appearance of ligated loops of small intestine from rabbits 24 h after injection with cultures of *Bacterium coli* grown at pH 8.4. Upper loop injection with a culture of pathogenic *Bact. coli*; lower loop with non-pathogenic *Bact. coli*. Reproduced from reference 3 with permission.

De’s work was published in 1956^3^. The results of a representative positive RIL is shown in Fig. 1. Unfortunately, this paper was “hidden” in the medical literature of the day; at that time there was no easy way to search for specific areas of research. (We did not know of its existence until after publishing our first papers on ETEC).

In 1959 De, using the RIL, was the first to discover cholera enterotoxin and to publish a book “Cholera, its Pathology and Pathogenesis^4^. The RIL developed by De was used in several laboratories for the study of enteropathogenic serotypes of *E. coli* from children with diarrhoea living in the developed world^5^. The results were equivocal, and no one other than De had examined *E. coli* from adults with the non-vibrio cholera syndrome.

## Clinical studies during a non-vibrio cholera epidemic

During the spring of 1963 an epidemic of non-vibrio cholera occurred in Kolkata, India and Dhaka, Bangladesh (then East Pakistan) which provided us with the opportunity to study these patients clinically and physiologically^6^,^7^. These detailed observations showed that the concentrations of electrolytes in the blood and stools were nearly identical to those in patients with *V. cholerae* induced cholera. The intravenous fluid used to effectively treat cholera was equally effective in these patients.

The clinical picture of the patients with non-vibrio cholera-like diarrhoea, and their details compared to those with cholera, is shown in the Table . It was clear that patients with non-vibrio cholera appeared in the hospital with the same symptoms of cholera, but their illness was of much shorter duration and required less fluid replacement. It should be noted that although tetracycline reduced stool output in patients with cholera, this response was not seen in those with non-vibrio cholera. Unfortunately, because we were not aware of De’s previous studies of *E. coli*, we did not do any tests on the large concentration of *E. coli* seen in the stool of these patients. As will be seen later, the non-vibrio cholera patients that we studied in 1968 had the same clinical picture as those studied in 1963, but without bacteriological confirmation of *E. coli* being involved.

**Table:** Comparison of clinical characteristics in 97 patients with cholera or non-vibrio cholera

Clinical characteristics	Cholera (n = 11)	Non-vibrio cholera (n = 86)
iv fluids needed (l)	17	5
Stool volumes (l)	16	2
Length of illness (days)	4-5	2
Response to tetracycline	+	-
*Source*: Data extracted from Ref. 6

In 1965, the first conference of the new U.S.-Japan Cooperative Science Program was held in Honolulu, Hawaii^8^. Some of us from Johns Hopkins University were at the meeting, and noted that many papers were presented that used the RIL in studies of *V. cholerae*. Unfortunately De was not able to attend and there were no papers on the testing of *E. coli* by the RIL at this time. Fortunately at that meeting, I met Dr William Burrows, who later sent a colleague of his to teach me a modified RIL, which I later used in Kolkata.

## The discovery of enterotoxigenic *E. coli*

In 1968, I was part of a team of four clinical scientists from the Johns Hopkins University School of Medicine who came to Kolkata as part of an International Center for Medical Research and Training (ICMRT), funded by the United States National Institutes of Health to study cholera, at the Calcutta School of Tropical Medicine and the Infectious Diseases Hospital. The cholera and ETEC team included Drs R. Bradley Sack, Sherwood Gorbach, John Banwell and Nathaniel Pierce from the Johns Hopkins University Center for Medical Research and Training, Baltimore, MD; Drs Benedicta Jacobs and B.D. Chetterjee from the Calcutta School of Tropical Medicine; and Drs Rupak Mitra and A. Mondal from the Infections Diseases Hospital, Calcutta (now Kolkata) during 1967-1970. Dr Sherwood Gorbach came to examine the microbiologic flora of the intestinal tract in patients with cholera. Dr John Banwell and Dr Nathaniel Pierce came to study the fluid and electrolyte movement in the small bowel of patients with cholera and their therapeutic implications, using intubation techniques. I came with the RIL to study the action of cholera toxin.

**Fig. 2:**
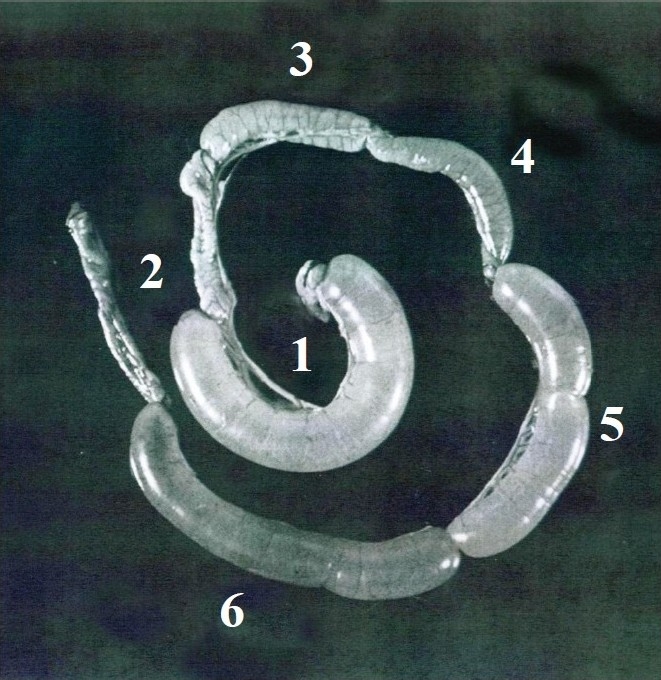
Titrations of *E. coli* culture filtrates in the RIL assay. Loops 1 and 2 represent positive (*V. cholerae* enterotoxin) and negative (saline) controls, respectively. Loops 6, 5, 4, and 3 show results obtained with increasing dilutions of the ETEC culture filtrates. Done in author’s laboratory.

Our study patients were adults admitted to the Infectious Disease Hospital with watery stool and severe dehydration and a presumptive diagnosis of cholera. Our research team got “side-tracked” because of the unexpected finding of seeing large concentrations of *E. coli* rather than *V. cholerae* in the small bowel of many of these patients thought to have cholera. It soon became clear that we were seeing many cholera patients who had only *E. coli* in their small bowel and stool. Small bowel samples were found to have a pure growth of *E. coli*, with concentrations of 10^6^ to 10^8^colony forming units (cfu) per ml. Stool samples also showed a predominance of *E. coli* at similar concentrations. This strongly suggested that *E. coli* may be an aetiologic agent of this syndrome. We immediately used the RIL test to determine whether they caused distention of the ileal loops, not only as live cultures but as culture filtrates. We were very excited to find that loops inoculated with either live cultures or culture filtrates caused distention of the loops, in the same way as cholera toxin was known to do. Furthermore the filtrates could be diluted up to 1:100 and still produce a positive RIL. The *E. coli* “diarrhoea factor” was quickly characterized and found to be non-dialyzable, heat-labile (when heated to 56°C for 30 min) and precipitated with 40 per cent ammonium sulphate. A picture of the positive RIL is shown in Fig. 2.

These data were first presented in November, 1968 at the US-Japan Cooperative Medical Science program in Unzen, Japan^9^. During the discussion (which was printed in those early days of the program) investigators from both the US and Japan indicated that they had examined enteropathogenic serotypes of *E. coli* from local children with diarrhoea, and found them to be negative^9^. At that time no one had tested *E. coli* from patients with severe cholera-like syndromes. Clinically, these patients harbouring enterotoxin-producing *E. coli* (ETEC) had the same findings as those studied in 1963. They had an abrupt onset of rice-water stools, and rapid onset of severe dehydration. Their illness lasted only 18 to 36 h with a low volume of stool, compared to cholera patients. They responded well to both intravenous and oral rehydration therapy. They had the same alteration in fluid and electrolyte movements in the small bowel, similar to patients with cholera.

Initially, the *E. coli* enterotoxin was produced using methods that resulted in toxin production by *V. cholerae*: i.e., growing the organisms in Syncase media in shallow culture flasks for 48 h, followed by centrifugation, filtering through a 0.22 μ Millipore filter, dialyzing for 3 days, lyophilizing and storing at 5°C.

Clearly, serotyping of the *E. coli* strain was of great importance because of determining whether these ETEC were of similar serotypes and whether they were of the known enteropathogenic serotypes. In fact, the ETEC were found to be several different serogroups: the most frequent were O6, O15, O25, O78, and O126^10^,^11^. None was found to be an enteropathogenic serotype. Enterotoxin production by the ETEC was found to be stable after many passages. In fact, we have some of these strains in our laboratories after 41 years. Another strain, H1O4O7, first isolated in Bangladesh (then East Pakistan) in 1975 is presently being used in volunteer challenges during ETEC vaccine development. To determine whether enterotoxins from different strains of *E. coli* were identical, rabbits were immunized parenterally with a single crude enterotoxin preparation. The resultant serum antibodies neutralized crude preparations of toxin from several different *E. coli* strains, using the RIL, indicating the similarity of the enterotoxins^12^. Immunological studies were done on 29 patients who had either ETEC-induced diarrhoea or cholera^13^. Convalescent sera neutralized both enterotoxins to some degree (higher titres in patients with the homologous infections) indicating the immunologic similarity between the two toxins.

**Fig. 3:**
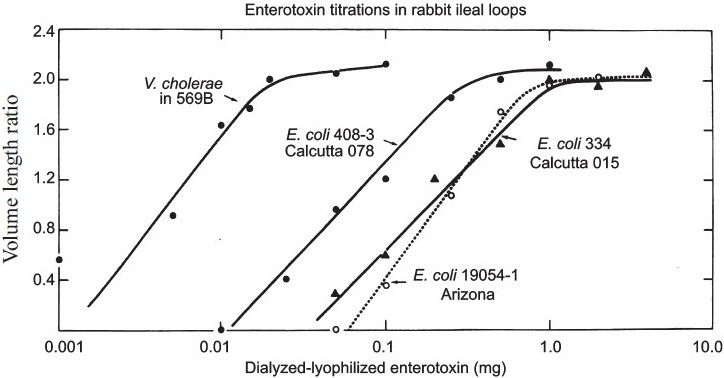
Titration of crude preparation of enterotoxin from three strains of ETEC and *V. cholerae* in the RIL. See reference 14 for details.

We compared the potency of the crude *E. coli* enterotoxin with a preparation of cholera toxin made by Wyeth Laboratories, and found it to be 1:10 to 1:100 as potent, using the RIL. A comparison of three preparations of crude *E. coli* enterotoxin with our own preparation of cholera toxin from strain *V. cholerae* 569B indicated the same differences in potency^14^ (Fig. 3.)

In September 1970 these data were presented at the US-Japan Cholera Conference in Yamanashi, Japan^15^. Twenty seven isolates of ETEC were obtained from 17 patients, one of whom was from the Cholera Hospital in Dhaka, Bangladesh (East Pakistan). In April, 1971, three papers describing our experience with the discovery of ETEC were published simultaneously^10^,^16^,^17^ (unfortunately, none of these papers included a reference to De’s 1956 studies indicating our unawareness of his studies). Data from the same 17 patients were reported in each of the three publications. Details of the ETEC enterotoxin were given by Sack *et al*^10^. Additional ETEC were described, along with the microbial flora of the entire intestinal tract in patients with cholera and ETEC- induced diarrhoea by Gorbach *et al*^16^. Fluid and electrolyte movement in the small bowel of patients with cholera or ETEC-induced diarrhoea were described by Banwell *et al*^17^.

**Fig. 4:**
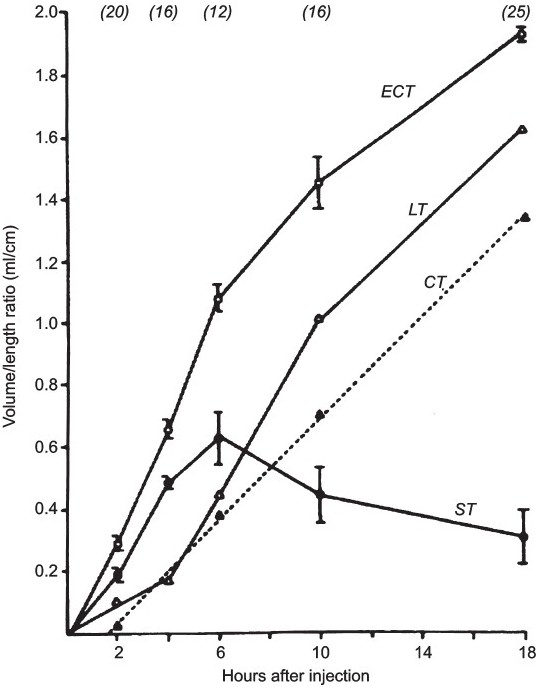
Production of fluid in the RIL at various times after inoculations with different enterotoxin preparations. ECT, crude *E. coli* enterotoxin; LT, heat-labile enterotoxin; ST, heat-stable enterotoxin; and the results are compared with that obtained with *V. cholerae* enterotoxin (CT). Reproduced from reference 18 with permission.

**Fig. 5:**
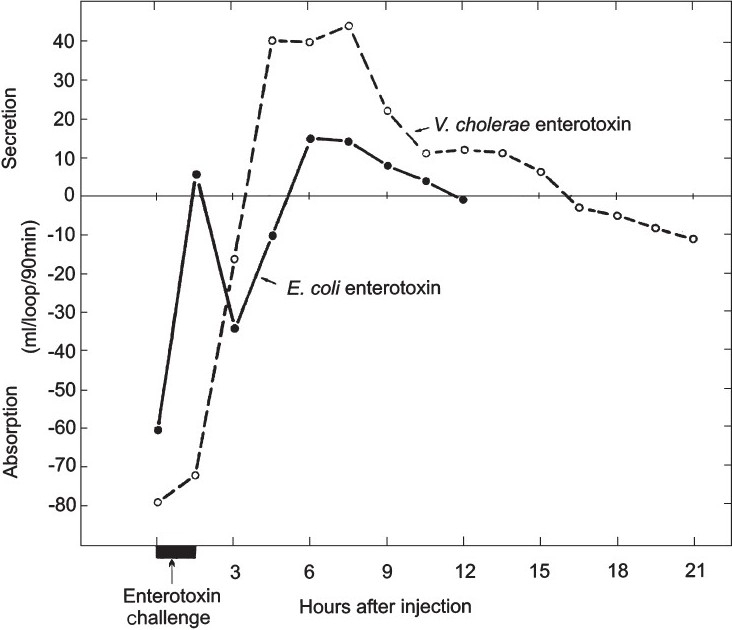
Fluid output from the small intestine using Thiry-Vella loops in dogs with either crude *E. coli* enterotoxin or *V. cholerae* enterotoxin. See references 14 and 19 for details.

Initially there were differences of opinion in the heat-stability of the crude enterotoxin preparations. Some investigators thought all preparations contained both heat-labile toxin (LT) and another heat-stable enterotoxin (ST). This was clarified by using other assays: the infant mouse assay was used to identify ST only and the RIL test was shown to measure a response to ST at 6 h and LT at 18 h^18^ (Fig. 4.) The Thiry-Vella loop assay in dogs clearly differentiated the time course of the secretory response to a crude *E. coli* enterotoxin preparation, showing an early and late response to ST and LT, respectively as compared to the late response of cholera toxin^19^ (Fig. 5.) Until about 1973 there was confusion about differentiating LT and ST. During these early days, only the RIL at 18 h was used, so that only LT would be detected. These questions were clarified when *E. coli* strains were found that produced only LT or ST.

**Fig. 6:**
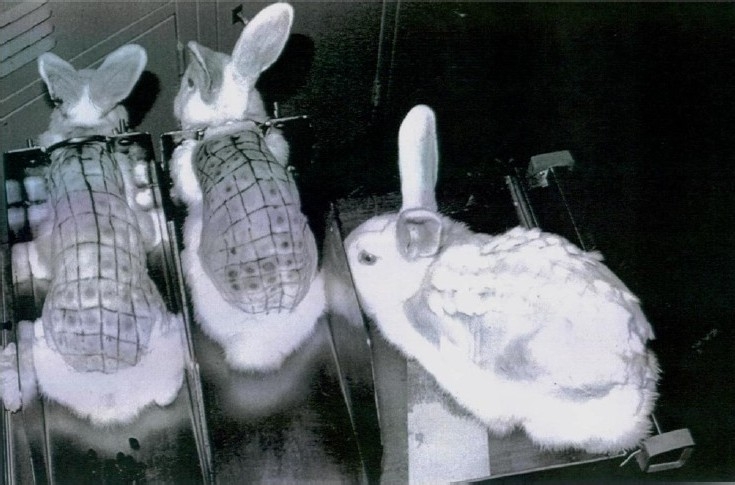
Titration of *V. cholerae* or *E. coli* enterotoxins by using the permeability factor assay in rabbit skin. The rabbit on the right had been injected about one month earlier and had rapid re-growth of hair at the site of injections. Done in author’s laboratory.

One additional assay should be mentioned, the vascular permeability factor. This factor is assayed in guinea pig or rabbit skin^20^ (Fig 6). It was known to be produced by *V. cholerae*, and could be used for assaying toxin or antitoxins. The same property was seen in heat labile preparations of *E. coli* enterotoxin. This was used in early experiments as an assay for LT. (Interestingly, it can be seen that hair growth occurred much more rapidly in the spots where the enterotoxin had been injected).

Early attempts to purify the LT enterotoxin were made using specific ganglioside agarose affinity columns, and also affinity columns using specific anti-cholera toxin antibodies. One of these investigators was Peter Agre, who was a medical student at Johns Hopkins School of Medicine at the time^21^ and later became a Nobel Laureate in 2003 for the discovery of aquaporins.

## *E. coli* enterotoxins in animals

Beginning in 1967, veterinary scientists examined *E. coli* isolates from young animals, particularly pigs, using the ileal loops of pigs, dogs, and rabbits^22^–^24^. During the next few years, they were able to identify enterotoxins (LT and ST) and colonization factors carried in plasmids of *E. coli* from these young animals with diarrhoea. It should be pointed out that although the enterotoxins from animal strains are closely related or identical to those from humans, animal strains of ETEC do not infect humans, this primarily due to the differences in colonization factors.

## Subsequent studies with ETEC

**Fig. 7:**
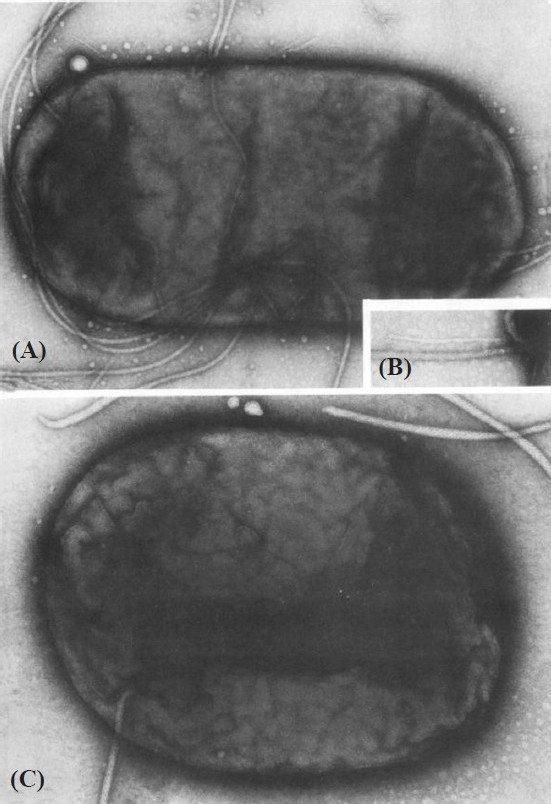
Photomicrograph of *E. coli* (ETEC) showing fimbria/pili **(A)**. × 60,000. The inset **(B)** shows a thin filament lying next to a flagellum. × 100,000. Flagella **(C)** as the only visible surface structure. × 100,000. Reproduced from reference 30 with permission.
- ETEC as a cause of “Travellers’ Diarrhoea” (TD)
  In 1970, an outbreak of TD in Aden was investigated by Rowe and colleagues^26^ who found a predominance of a single serotype of *E.coli*, (O148:H28). Unfortunately, testing for enterotoxins was not available at that time. One year later in England, a laboratory technician working with this strain developed severe diarrhoea from which this same serotype was isolated. Furthermore, one of the strains used in the volunteer studies had the same serotype that was isolated from soldiers in Vietnam and was found to produce LT and a cholera-like syndrome in the volunteers^26^. Although not completely documented this appears to be the first recognized outbreak of TD caused by ETEC. In 1974, Shore and colleagues^32^ studied 28 travellers from Boston who had visited developing countries. Eleven developed TD, of which 4 (14%) had ETEC isolated from stool specimens. The assay used to detect ETEC was the infant mouse technique, so that only ST-producing *E. coli* could be recognized. In 1975, Gorbach and colleagues^33^ studied American students travelling to Mexico. Thirty eight of 123 (29%) developed TD. ETEC were found in 72 per cent of those with TD and in 15 per cent of students who were healthy. The assay used for detecting ETEC was the rabbit skin permeability factor which detects LT. No assays for ST were done. This study demonstrates the great importance of LT-producing *E. coli* in the aetiology of TD. In 1976, Merson and colleagues^34^ studied a group of 73 physicians and their families visiting Mexico to attend a Gastroenterology Conference. Of the 107 travellers, 59 (49%) developed TD; ETEC was isolated from 21 (36%) of those with TD. The methods used to detect ETEC were the adrenal cell assay, the Chinese hamster ovarian cell assay, the infant mouse and the RIL. In this study both LT and ST-producing *E. coli* were identified. Since that time there has been a large number of published studies verifying the role of ETEC as the most frequent cause of TD.
- Diagnosis of ETEC diarrhoea and cholera
  ETEC diarrhoea is less severe than cholera, but may present exactly the same early clinical appearance. ETEC diarrhoea is relatively short-lived and requires less fluid replacement. Cholera can be treated effectively with antibiotics while ETEC diarrhoea has not clearly been shown to respond to antibiotics in endemic setting. In later studies antibiotics are clearly shown to be effective in the treatment of TD. ETEC has no phenotypic characteristics to make it recognizable in a plate of *E. coli*. *V. cholerae* can easily be recognized with the use of inhibitory media, such as thiosulphate citrate bile salts sucrose (TCBS) agar. For these reasons ETEC cannot be detected in minimally-equipped laboratories, whereas cholera can be easily diagnosed. As a result there have been relatively a few epidemiologic studies of ETEC diarrhoea.
- Clinical understanding of ETEC diarrhoea in 2009
  ETEC are the most common bacterial cause of diarrhoea in children living in the developing world. It is estimated that as many as 500,000 children may die annually. ETEC are the most frequent cause of TD among travellers from developed countries to developing countries. Thus association must be clear. Travellers are much like children living in those areas; both groups are immunologically naive and therefore lacking in resistance to infection by ETEC^35^. The most important ongoing studies of ETEC are: (*i*) developing an effective, low-cost vaccine that can be used in children living in the developing world, as well as tourists visiting these areas, (*ii*) configuration of ST to make it antigenic, and (*iii*) developing a simpler test to identify ST. (The many rapid genetic tests for ETEC are available only in limited areas).

- The first volunteer challenge studies with ETEC
  This was reported in 1971 by DuPont and colleagues^25^, using two *E. coli* strains obtained from two American soldiers in Vietnam who had been diagnosed with “colitis”, probably similar to what we know as travellers’ diarrhoea. They did not have a cholera-like illness. These strains were found to produce LT, using the RIL. One of the strains (O148H28) was antigenically similar to the strain that had been identified in 1970 during an outbreak of travellers’ diarrhoea among 19 of 38 British soldiers deployed in Aden^26^.
  The study was done in healthy volunteers at the Maryland House of Correction. At high doses of inoculum (1×10^10^cfu), 7 of 10 volunteers developed a cholera-like illness and an antibody response to LT was seen in 6 volunteers. (Many years later volunteers’ studies were done with *E. coli* that produced either LT or ST or both enterotoxins).
- ETEC diarrhoea in the United States
  In 1972 a search was made for ETEC in the stools of children hospitalized with diarrhoea at Cook County Hospital, Chicago^27^. Studies were done in 29 children with diarrhoea in whom no other enteric pathogen could be isolated. Using the Infant Rabbit Test, 24 of these children (83%) were positive. This high rate of positive results was not found in other studies, and is probably related to variations in the ETEC assays. In 1973, my colleagues and I^28^ conducted a search for ETEC in 59 children with acute diarrhoea who were under 4 years of age, living in the White Mountain Apache Tribe in Arizona, an area known to have high rates of diarrhoea in children. The assays used to detect ETEC were: RIL, infant rabbits, infant mice, and an adrenal cell assay. Eleven children had small bowel intubation performed in addition to routine stool cultures. The number of children with LT-producing ETEC in their stool was 10/59 (17%). No ST-producing ETEC was found. All of the intubated children had high concentrations of ETEC (10^3^to 10^9^cfu) in the small bowel. Antibodies to LT, using the adrenal cell assay, were studied in this general population and an increase in level of these antibodies with age was found^29^. These data demonstrate the importance of ETEC as a cause of diarrhoea in this population.
- The discovery of pili (fimbria) in ETEC in 1975
  Pili have been known to occur in *E. coli* for many years. In 1955, Duguid and his colleagues^30^ found “non-flagellar filamentous appendages (fimbria) and haemaglutination activity in *Bacterium coli*.” These *E. coli* were not particularly related to illness, but were clearly demonstrated by electron microscopy^30^.
  In 1975, a colonization factor was first identified on ETEC by Evans and colleagues^31^ (Fig 7). It was found to be a transmissible surface-associated colonization factor (pili), which was shown to be a critically important virulence factor. *E. coli* strain H10407 was isolated from a woman with cholera-like diarrhoea hospitalized in the Cholera Hospital in Dhaka, Bangladesh. This strain and an identical strain without fimbria were tested in infant rabbits; only the fimbriated strain was virulent. (Many later studies also clearly showed the importance of fimbria in the virulence of ETEC.) Today there are at least 23 ETEC colonization factors that are antigenically distinct. A colonization factor was not recognized in *V. cholerae* until about 10 yr later.

## Conclusion

Nine years after the discovery of cholera enterotoxin by S.N. De in 1959^3^ the discovery of enterotoxigenic *E. coli* in 1968^9^ in patients with non-vibrio cholera was another major milestone in our understanding of aetiologies of acute infectious diarrhoeal diseases. From initial studies using rabbit ileal loops, the two enterotoxins of *E. coli* have now been identified at the molecular level, and pili, the other critical virulence factor, has been identified and now is known to consist of at least 23 different antigenic types. In the last 42 years ETEC has been extensively studied, microbiologically, clinically, genetically and epidemiologically. It is now known that ETEC are the most common bacterial agents of diarrhoea of children living in the developing world, and also the most common cause of “Travellers’ diarrhoea” among visitors from the developed world travelling to the developing world. Although the improvement in water and sanitation is the ultimate long term goal for prevention of diarrhoeal illness, in the near future the development of an oral ETEC vaccine as a short term solution should be possible.
